# CCDC154 Mutant Caused Abnormal Remodeling of the Otic Capsule and Hearing Loss in Mice

**DOI:** 10.3389/fcell.2021.637011

**Published:** 2021-02-04

**Authors:** Kai Xu, Xue Bai, Sen Chen, Le Xie, Yue Qiu, He Li, Yu Sun

**Affiliations:** ^1^Department of Otorhinolaryngology, Union Hospital, Tongji Medical College, Huazhong University of Science and Technology, Wuhan, China; ^2^Department of Otolaryngology, The First Affiliated Hospital of Wenzhou Medical University, Wenzhou, China

**Keywords:** CCDC154, otosclerosis, hearing loss, auditory ossicles, otic capsule

## Abstract

Osteopetrosis is a rare inherited bone disease characterized by dysfunction of osteoclasts, causing impaired bone resorption and remodeling, which ultimately leads to increased bone mass and density. Hearing loss is one of the most common complications of osteopetrosis. However, the etiology and pathogenesis of auditory damage still need to be explored. In this study, we found that a spontaneous mutation of coiled-coil domain-containing 154 (CCDC154) gene, a new osteopetrosis-related gene, induced congenital deafness in mice. Homozygous mutant mice showed moderate to severe hearing loss, while heterozygous or wild-type (WT) littermates displayed normal hearing. Pathological observation showed that abnormal bony remodeling of the otic capsule, characterized by increased vascularization and multiple cavitary lesions, was found in homozygous mutant mice. Normal structure of the organ of Corti and no substantial hair cell or spiral ganglion neuron loss was observed in homozygous mutant mice. Our results indicate that mutation of the osteopetrosis-related gene CCDC154 can induce syndromic hereditary deafness in mice. Bony remodeling disorders of the auditory ossicles and otic capsule are involved in the hearing loss caused by CDCC154 mutation.

## Introduction

Osteopetrosis is a rare bone disorder caused by the absence or dysfunction of osteoclasts. This leads to a marked increase in bone density due to defective bone resorption (Del Fattore et al., 2008). Osteoclast-mediated bone resorption plays a vital role in bone homeostasis, and perturbation of this process can lead to profound alterations in bone mass that have clinical relevance. In addition to skeletal lesions, patients with osteopetrosis are often also affected by neurological complications, notably hearing loss or visual impairment. Approximately 80% of patients with osteosclerosis develop hearing loss in childhood. Most deaf patients present with conductive deafness due to abnormalities of the auditory ossicles or temporal bone, while some present with sensorineural or mixed hearing loss (Stocks et al., 1998; Dozier et al., 2005). Most cases of osteosclerosis are caused by genetic mutations, but a few cases still lack an accurate molecular diagnosis (Sobacchi et al., 2013). These findings suggest that osteosclerosis-related genes are also involved in the formation and maintenance of normal hearing.

There are many animal models of osteosclerosis with hearing-related symptoms. The osteopetrotic mutation toothless (tl) rat exhibits auditory ossicle abnormalities and hearing loss due to the truncated *Csf1* gene (encoding colony-stimulating facter-1, CSF-1) and non-functional protein (Aharinejad et al., 1999; Van Wesenbeeck et al., 2002). Auditory brainstem and visual evoked potentials are both abnormal in CSF-1-deficient mice (Michaelson et al., 1996). Osteoprotegerin (OPG) is a key regulator of bone homeostasis. Mice lacking OPG show abnormal remodeling of the otic capsule and auditory ossicles induced by osteoclast hyperactivity (Zehnder et al., 2005; Kanzaki et al., 2006). Moreover, the absence of OPG in the inner ear causes demyelination of the cochlear nerve and sensorineural hearing loss (Kao et al., 2013).

The mammalian middle ear contains the most delicate bone structure, called the ossicular chain, which consists of the malleus, incus, and stapes. These ossicles transmit vibrations from the tympanic membrane through the oval window to the inner ear, where the vibrations are converted into electrical signals in the otic capsule of the temporal bone and transduced to the brain *via* auditory nerves (Frolenkov et al., 2004). Osteoclasts are essential for development of the bone structures of the middle ear (Mallo, 2001), and the absence of osteoclastic resorption perturbs the process of bone resorption in the auditory ossicles or the otic capsule, thus significantly affecting their morphology and function (Kanzaki et al., 2011). Therefore, abnormal osteoclasts may cause deafness by affecting the bone structure development of the middle and inner ears. A new spontaneous autosomal recessive osteopetrosis mouse strain was reported by Lu et al. (2009). A CCDC15 mutant was identified in this strain and the osteoclasts were deficient in bone resorption. These mice displayed an osteopetrotic phenotype, including lack of tooth roots, relatively pyknotic and much thinner cortical bone. However, whether the mutant mice had auditory system complications had not been explored. In this study, we evaluated hearing function in this mouse line. Furthermore, the pathology of the auditory ossicles and the inner ear was investigated. Abnormal bony remodeling due to disordered osteoclastic bone resorption was observed in the otic capsule of homozygous mutant mice which displayed congenital deafness with abnormal bony remodeling in the otic capsule and auditory ossicles. However, the morphology of the organ of Corti (OC) showed normal organization and there was no cell loss in the auditory sensory epithelium or the spiral ganglion neurons (SGNs). Our findings provide further support for a critical role of osteoclasts in the development of auditory ossicles and the otic capsule. Abnormal bony remodeling of the auditory ossicles and otic capsule due to deficient osteoclasts might be the potential cause of hearing loss in CCDC154 mutant mice.

## Materials and Methods

### Mouse Model

Osteopetrosis mutant (ntl) mice were provided by Prof. Xin-Cheng Lu at Wenzhou Medical College. Homozygous mutant mice were generated by crossbreeding the heterozygous mutant mice. As reported previously (Lu et al., 2009; Liao et al., 2012), mouse genotyping was performed by PCR amplification of tail genomic DNA, using the following genotyping primers:

CCDC154-mutant: (F) -5′CAGTCATGGCAATGACAAA CA-3′
CCDC154-mutant: (R) -5′CAGGAAGGACCTAGCAAG ATA-3′
CCDC154-wild-type: (F)-5′TGGGGTGGGAGACTGGTT ATGTGT-3′
CCDC154-wild-type: (R)-5′GTGGGGCCGCAGTTGTC AGAAG-3′.

All mice were raised in the specific-pathogen-free Experimental Animal Center of Huazhong University of Science and Technology. All experimental procedures were conducted in accordance with the policies of the Committee on Animal Research of Tongji Medical College, Huazhong University of Science and Technology.

### Auditory Brainstem Response

Auditory brainstem response (ABR) was examined at P20. As we previously reported (Chen et al., 2018), mice (*n* = 5 in each group) were anesthetized by intraperitoneal injection with a mixture of ketamine (120 mg/kg) and chlorpromazine (20 mg/kg). Body temperature was maintained by placing the mice on an electric blanket. The recording electrode was placed at the vertex of the skull, and the reference electrode was placed at the tested ear, with an earth electrode placed at the contralateral ear. Tone bursts of 8, 16, 24, 32, and 40 kHz were generated and responses were recorded using a Tucker-Davis Technologies system (RZ6, Tucker-Davis Tech., Alachua, FL, United States). The responses were recorded as the average response to 1,024 stimuli and were recorded in decreasing 10 dB steps, which narrowed to 5 dB steps near the threshold. The lowest sound level that could be recognized was considered to be the auditory threshold.

### Preparation and Morphological Examination of Auditory Ossicles

Mice were deeply anesthetized and then culled by cervical dislocation. The middle ear was exposed by dissection of the bulla, and then the malleus, incus, and stapes were carefully separated from the middle ear. The collected tissues were fixed in 4% paraformaldehyde at room temperature for 2 h. For frozen sections, after decalcification with disodium EDTA for 48 h, the auditory ossicles were dehydrated with sucrose and embedded in OCT overnight at 4°C. Sections with a thickness of 10 μm were cut for morphological examination. Hematoxylin-eosin (HE) staining was performed following standard protocols.

### Cochlear Tissue Preparation and Immunofluorescent Labeling

Mice were deeply anesthetized and sacrificed at P20. The cochleae were carefully dissected from the temporal bones and fixed in 4% paraformaldehyde at room temperature for 1 h. For frozen sections, after decalcification with disodium EDTA for 48 h, the cochleae were dehydrated in 20 and 30% sucrose for 1.5 h each and then embedded in OCT overnight at 4°C. Modiolar sections with a thickness of 10 μm were cut for subsequent procedures as described previously (Zhou et al., 2016). For flattened cochlear preparations, each stretched cochlear preparation was carefully dissected from decalcifying cochleae in PBS. The sections or flattened cochlear preparations were incubated in a blocking solution of 10% donkey serum with 0.1% Triton X-100 for 1 h at room temperature, and then incubated with polyclonal rabbit anti-myosin 7a antibodies (1:500 dilution, 25–6,790, Proteus Bio-Sciences, Ramona, CA, United States), or polyclonal goat anti-sox2 antibodies (1:200 dilution, AF2018, R&D systems, Minneapolis, MN, United States) diluted in PBS with 0.3% Triton X-100 overnight at 4°C. Samples were washed three times in PBS with 0.1% Tween-20 and then stained with Alexa Fluor 647 donkey anti-goat IgG or Alexa Fluor 488 donkey anti-rabbit IgG (1:200 dilution; ANT032 and ANT031, Antgene Biotechnology Company Ltd., Wuhan, China) for 2 h at room temperature. DAPI (C1005; Beyotime Biotechnology) and phalloidin (0.05 mg/mL; P5282; Sigma-Aldrich, St. Louis, MO, United States) were used for nuclear and F-actin staining. Images of each cochlea turn were obtained with a laser scanning confocal 408 microscope (Nikon, Tokyo, Japan).

### Nissl Staining Analysis

Animals were deeply anesthetized and heart perfusion was performed with 4% paraformaldehyde in PBS. The brains were carefully removed and fixed in 4% paraformaldehyde overnight at room temperature, and then dehydrated sequentially through graded alcohol, and embedded in paraffin following a conventional protocol. Sections with a thickness of 5 μm were cut for Nissl staining. Transverse sections were deparaffinized with xylene, followed by rehydration in graded alcohol and immersion in 0.3% toluidine blue for 40 min at 60°C as described previously (Chen et al., 2015). The number of neurons in the V layer of the auditory cortex was counted.

### Data Analyses

All data are presented as means ± S.D. and were plotted by GraphPad Prism (Version 8.0, GraphPad Software Inc., La Jolla, CA, United States). The t-tests were performed using SPSS software (version 19, IBM SPSS Statistics, Armonk, NY, United States), and *P* < 0.05 was considered to be statistically significant.

## Results

### Edentulism and Significant Hearing Loss Was Observed in the Homozygous Mutant Mice

**FIGURE 1:**
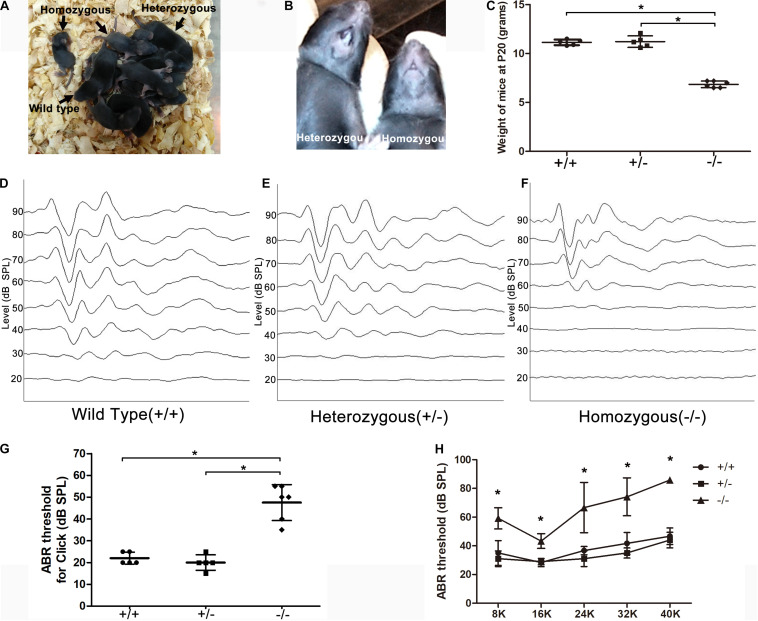
Significant hearing loss was observed in the homozygous mutant mice. **(A)** A litter of CDCC mutant mice, homozygous mutant mice showed smaller body size compared to wild-type or heterozygous littermates. **(B)** Homozygous mutant mice displayed no tooth. **(C)** Comparison of the weight at P20 between the three groups **(D–F)** ABR-click waveforms in wild type **(D)**, heterozygous **(E)**, and homozygous mice **(F)**, respectively. **(G)** Comparison of the ABR-click thresholds between the three groups. **(H)** Comparison of tone-burst thresholds in the three groups. *Significantly different from the control group (*P* < 0.05). All ABR tests were performed at P20.

The homozygous mutant mice had a smaller body size compared to wild-type (WT) or heterozygous littermates (Figure 1A) and no tooth eruption (Figure 1B). The homozygous mutant mice weighed only approximately half as much as the WT or heterozygous mutant mice at P20 (Figure 1C). ABR was tested at P18–20. The homozygous mutant mice showed hearing loss at all frequencies, while heterozygous or WT littermates displayed normal hearing (*n* = 5 mice in each group). The different ABR-click waveforms in the three groups are shown (Figures 1D–F). The minimum sound intensity to evoke a response (threshold) was 50.0 ± 5.5 dB sound pressure level (SPL) in the homozygous mutant mice and 22.5 ± 2.5 or 20.0 ± 3.2 in the heterozygous or WT mice, respectively (Figure 1G, *P* < 0.05). ABR-click waveforms showed that there was obvious wave I–III at 70–90 dB SPL in homozygous mutant mice (Figure 1F). The thresholds of homozygous mutant mice at 8, 16, 24, 32, and 40 kHz were 62.0 ± 2.4, 44.0 ± 14.9, 72.0 ± 11.7, 79.0 ± 5.8, and 86.0 ± 2 dB SPL, respectively. In comparison, the hearing thresholds of WT mice were 33.8 ± 6.5, 27.5 ± 2.5, 35.0 ± 3.5, 41.3 ± 5.4, and 47.5 ± 4.3 dB SPL respectively, at corresponding frequencies. Differences between the homozygous and WT animals were significant at all frequencies (Figure 1H, *P* < 0.05, one-way ANOVA).

### Abnormal Structure of the Auditory Ossicles and Otic Capsule in the Mutant Mice

**FIGURE 2:**
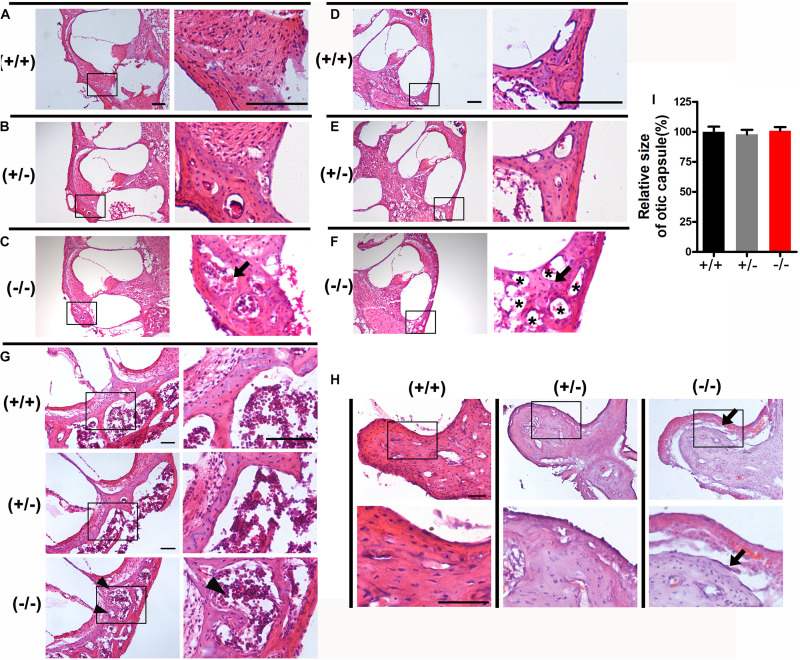
Histological examination of the otic capsule and auditory ossicles in the mutant mice. **(A–C)** The basal turn of modiolar sections stained with hematoxylin-eosin (HE) in wild type **(A)**, heterozygous **(B)**, and homozygous **(C)** mutant mice, respectively. **(D–F)** The middle turn of otic capsule in wild type **(D)**, heterozygous **(E)**, and homozygous **(F)** mutant mice, respectively. Black arrows indicate the regions of abnormal bone remodeling in otic capsule from homozygous mutant mice **(C,F)**, Asterisks indicate the numerous large vascular channels **(F)**. **(G)** The apical turn of otic capsule in the three groups. Black arrowheads indicate the regions of abnormal bone remodeling and erodes the cartilage of otic capsule. **(H)** Representative images of malleus stained with hematoxylin-eosin (HE) from three groups. Black arrows indicate abnormal bone resorption and the bony cortex of the malleus was thickened in homozygous mutant mice. **(I)** Relative heights of the organ of Corti in the wild type and mutant mice. The scales in panels **(A,D,G,H)** represent 100 μm.

Disruption of transmission in the middle ear can also be associated with elevated ABR thresholds. The tympanic membrane and middle ear of mice were observed under an optical microscope after posterior auricular incision. The tympanic membrane and bulla were normal, there was no effusion in the tympanum and no evidence of infection was seen in mutant mice. To examine the morphology of the auditory ossicles, we isolated the malleus, incus, and stapes from the middle ear cavities. There were no significant structural differences of the malleus, incus or stapes in homozygous mutant mice compared with heterozygous or WT littermates. To further analyze the histological characteristics of the auditory ossicles and otic capsule in mutant mice, sections of auditory ossicles stained with HE showed increased active bone remodeling of the otic capsule in homozygous mutant mice compared with heterozygous or WT littermates. Notable features of the active bone remodeling included formation of well-defined hypercellular areas, abundant angiogenesis and cavitary lesions in the otic capsule showing bone resorption and deposition (Figures 2C,F), compared to the same area in the heterozygous or wild type (Figures 2A,B,D,E). Abnormal bone resorption and hypercellular erosion of the cartilage of the otic capsule in the apical turn were observed in homozygous mutant mice, while the cartilage in heterozygous or WT mice displayed intact boundaries (Figure 2G). However, the size of ear cartilage capsule of homozygous mutant mice was not significantly different, compared to heterozygous and WT mice (Figure 2I). Furthermore, the auditory ossicles also showed remodeling, as evidenced by abnormal bone resorption, and the bony cortex of the malleus was thickened in homozygous mutant mice (Figure 2H).

### Normal Structure of the Organ of Corti and no Hair Cell Loss Were Observed in the Mutant Mice

**FIGURE 3:**
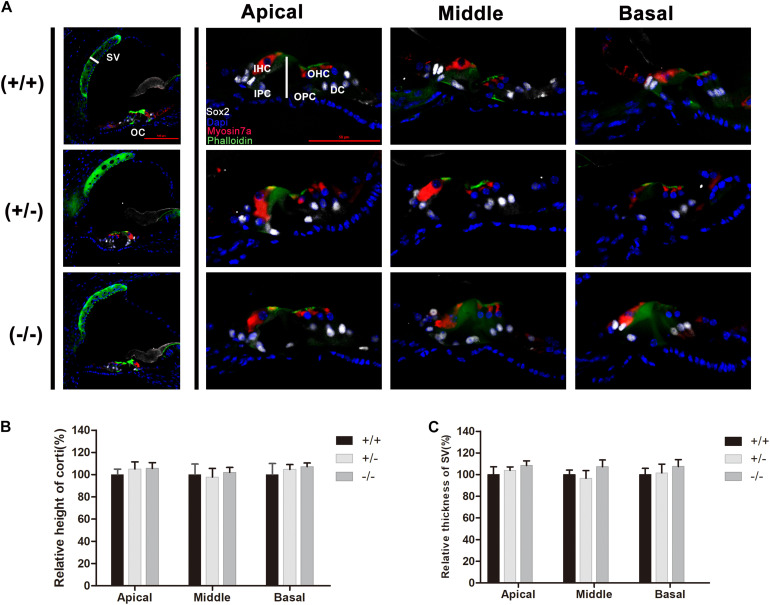
There was no significant change in the cochlear morphology of the mutant mice. **(A)** Myosin7a (red) and Sox2 (white) immunolabeling showing the morphology of the organ of Corti and spiral ligament in different turns from the wild type, heterozygous and homozygous mutant mice. **(B)** Relative heights of the organ of Corti in the wild type and mutant mice. **(C)** Relative thickness of the organ of Corti in the wild type and mutant mice. The scales in panel **(A)** represent 50 μm.

**FIGURE 4:**
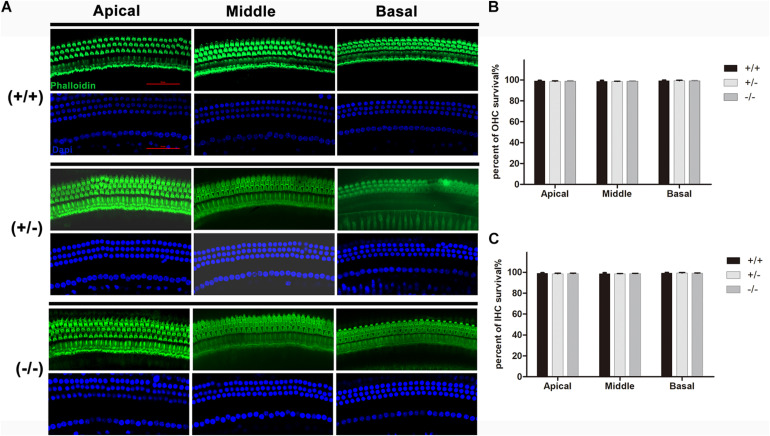
There was no significant degeneration of hair cell in the mutant mice at P20. **(A)** Representative images of HCs (Phalloidin, green) in the apical, middle, and basal turn of basilar membrane from the wild type, heterozygous and homozygous mutant mice. **(B)** Quantifications of OHCs survival at specific cochlear locations in the different groups at P20. **(C)** Quantifications of IHCs survival at specific cochlear locations in the different groups at P20. The scales in panel **(A)** represent 50 μm.

Normal formation of the OC is essential for hearing development, and the tunnel of Corti usually opens completely in all turns at P8–P9 (Roth and Bruns, 1992a,b). Immunostaining of radial sections showed that the tunnel of Corti and the spiral ligament were well formed at P20 in homozygous mutant mice (Figure 3A). In mice from different groups, hair cells were labeled by Myonsin7a (red) while supporting cells expressed Sox2 (white Figure 3A). There was no significant change in the height of the OC in any of the three turns of homozygous mutant mice compared with WT mice (Figure 3B). The stria vascularis (SV) showed normal three-layered organization and the thickness was not significantly changed in homozygous mutant mice (Figures 3A,C). No substantial hair cell loss was observed in heterozygous or homozygous mutant mice at P20 (Figures 4A–C).

### There Was No Significant Degeneration of Spiral Ganglion Neurons or Auditory Cortical Neurons in Mutant Mice

**FIGURE 5:**
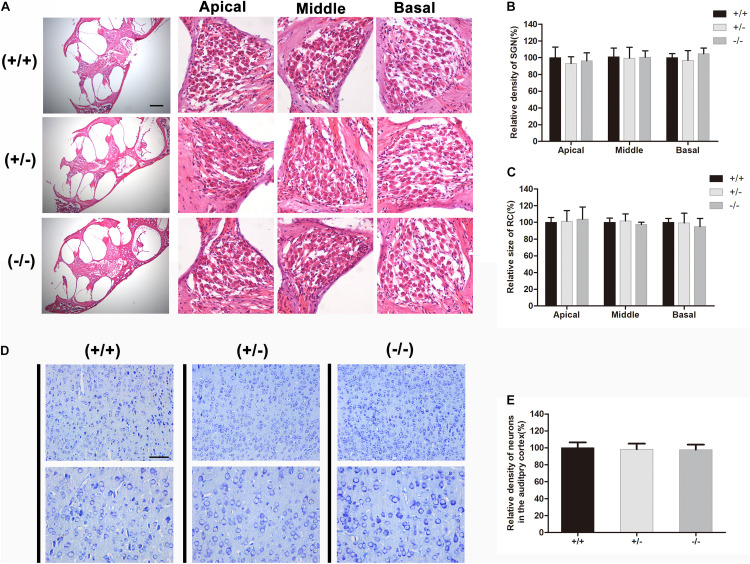
There was no obvious degeneration of spiral ganglion neuron or auditory cortical neurons in mutant mice at P20. **(A)** Full view of the cochlea and representative images of SGN in different turns from the wild type, heterozygous and homozygous mutant mice, respectively. **(B)** Quantifications of SGNs survival at specific cochlear locations in the different groups at P20. **(C)** Quantifications of RC size at specific cochlear locations in the different groups at P20. **(D)** Representative images of neurons in auditory cortical from wild type, heterozygous and homozygous mutant mice, respectively. **(E)** Quantifications of surviving neurons in the auditory cortical from different groups at P20. The scales in panels **(A,D)** represent 200 and 100 μm, respectively.

HE-stained radial sections were used in morphologic studies. A full view of the cochlea of wild type, heterozygous and homozygous mice were showed. The Rosenthal canal (RC) was amplified for further investigation (Figure 5A). After quantifying the area of the RC and the number of SGNs, no significant degeneration of the SGNs was observed in homozygous mutant mice, and no significant change in the area of the RC (Figures 5B,C). Counting the number of neurons stained by toluidine blue in the auditory cortex (Figure 5D) revealed that there was no significant change in the density of auditory cortex neurons in homozygous mutant mice compared with WT mice (Figure 5E).

## Discussion

The CCDC154 mutant mouse strain exhibits congenital deafness and skeletal abnormalities. This strain displays no tooth root formation but instead shows development of odontoma, a common feature of osteopetrosis, and the skeletal abnormalities are also closely similar to human osteopetrosis (Lu et al., 2009). Moreover, further work demonstrated that there was a ∼5 kb deletion comprising exons 1–6 of the CCDC154 gene in the mutant mice (Liao et al., 2012). However, whether the mutant mice had other osteosclerosis-related phenotypes has not been reported. In the present study, our data demonstrate that the homozygous mutant mice showed severe hearing loss at high frequency and moderate deafness at low frequency, while heterozygous or wild type littermates displayed normal hearing. These data suggest that the CCDC154 fragment deletion can cause syndromic hereditary deafness in mice.

To date, most research into hearing loss has focused on hair cells in the inner ear. Many genes are crucial for the development and survival of hair cells. Zhou et al. (2020) reported that genetic ablation of Atg7 in outer hair cells (OHCs) in mice caused stereocilium damage and electromotility disturbances, which led to the degeneration of OHCs and subsequent early-onset profound hearing loss. Disrupted function of slc4a2b resulted in a decreased number of HCs in zebrafish neuromasts due to increased HC apoptosis (Qian et al., 2020). Knockdown Arhgef6 in mice caused progressive hearing loss due to HC loss and stereocilia deficits (Zhu et al., 2018). Fang et al. (2019) reported that loss of Limk1 and Limk2 did not affect the overall development of the cochlea and the structure of hair bundles. CCDC154 is not necessary for the survival of hair cells or spiral ganglion cells. The morphology of the OC and the spiral ligament of the cochlea showed normal organization in all turns and there was no substantial hair cell or SGN degeneration in mutant mice. ABRs showed that there was obvious wave I–III at 70–90 dB SPL. These results prove that the inner ear of mutant mice can still transmit acoustic signals to the primary auditory nucleus under high stimulation. In the cuticular plate of hair cells, they are thought to be critical for mammalian hearing (Qi et al., 2019). CCDC154 mutant mice showed normal structure of hair bundles.

Fragment deletion in CDCC154 plays a vital role in bone remodeling of the otic capsule and auditory ossicles. The otic capsule is unique in its composition and pattern of bone remodeling; unlike most bones in the skull that form through intramembranous ossification, the auditory ossicles and otic capsule are formed through endochondral ossification (Tucker et al., 2004). During this process, the hypertrophic chondrocytes and calcified cartilage matrix are absorbed by osteoclasts and the cartilage template is subsequently replaced by bone (Mallo, 2001). The auditory ossicles or otic capsule are almost absent from bone remodeling after development, and the bone remodeling unit has a centrifugal distribution within the inner ear tissues (Frisch et al., 1998). Although osteoclastogenesis is normally suppressed in the ossicles and the auditory otic capsule, osteoclast function is still required to sculpt these bones during development. The reasons for this low level of bone turnover remain unclear. Studies have reported that high levels of OPG in the inner ear may inhibit bone remodeling in the otic capsule (Zehnder et al., 2005). In humans, a disturbed balance of OPG expression in the otic capsule is associated with otosclerosis, a complex bone dystrophy of the human otic capsule leading to conductive and sensorineural hearing loss (Karosi et al., 2011). The typical pathologic feature of otosclerosis is abnormal bony remodeling of the otic capsule, which includes osteoclast-mediated bone resorption and increased vascularization, osteoblast-mediated bone formation and new bone deposition (Quesnel et al., 2018). The active bone remodeling process of the otic capsule in the CCDC154 mutant mice was strikingly similar to that observed in the temporal bone of otosclerosis patients. Typical pathological features of the otic capsule in CDCC154 mutant mice include formation of well-defined hypercellular areas, increased vascularization and new bone or mineralization deposition, which resembles the lesions of active otosclerosis (Parahy and Linthicum, 1984; Quesnel et al., 2018). Zehnder et al. (2006) also observed similar abnormalities of bone remodeling of the otic capsule and hearing loss in OPG knockout mice. Our results suggest that CCDC154 is essential for normal bone remodeling of the otic capsule and auditory ossicles, which is important for maintaining normal auditory function. However, CCDC154 is a novel gene. To date, the function of the CDCC family has been poorly studied. Coiled-coil domain containing (CCDC) family members enhance tumor cell proliferation has been reported. Zhang et al. (2017) reported that CCDC106 promotes non-small cell lung cancer cell proliferation. Overexpression of a novel osteopetrosis-related gene CCDC154 suppresses cell proliferation by inducing G2/M arrest (Liao et al., 2012). Dong et al. (2019) reported that CCDC154 was key proteins involved in the molecular mechanisms of Parkinson’s disease (PD), which may be used as novel plasma biomarkers for early diagnosis of PD and the future development of treatments. However, its function in the middle ear or inner ear is unclear. Therefore, more studies are needed to explore the function of CCDC154 in the auditory system.

Otosclerosis is a disease of the bony labyrinth of the inner ear with a prevalence of 0.3–0.4% in the European population but which is rare among Asians and Africans (Declau et al., 2001). The abnormal bone remodeling of the otic capsule results in progressive conductive hearing loss, and up to one-third of patients ultimately develop sensorineural hearing loss in addition to conductive hearing loss (Ishai et al., 2016). However, the etiology of otosclerosis remains poorly understood. Both genetic and environmental factors such as estrogens, fluoride, and viral infection have been implicated in the disease process. To date, several otosclerosis loci named OTSC1–10 have been mapped in families showing segregation of autosomal dominant otosclerosis, although none of the otosclerosis-causing genetic mutations within these locations has been identified so far (Babcock and Liu, 2018). Our results suggest that the CDCC154 mutation may be associated with otosclerosis, even though the mutation has never been found in otosclerosis patients. In future this gene may be worthy of investigation in patients with otosclerosis, and the CDCC154 mutant mouse strain may provide a valuable animal model of human otosclerosis.

## Data Availability Statement

The original contributions presented in the study are included in the article/supplementary material, further inquiries can be directed to the corresponding author/s.

## Ethics Statement

The animal study was reviewed and approved by the Committee on Animal Research of Tongji Medical College, Huazhong University of Science and Technology. Written informed consent was obtained from the owners for the participation of their animals in this study.

## Author Contributions

YS and HL conceived and designed the study. KX, XB, SC, LX, and YQ performed the experiments. KX, XB, and SC wrote the manuscript. YS and HL reviewed and edited the manuscript. All authors read and approved the manuscript.

## Conflict of Interest

The authors declare that the research was conducted in the absence of any commercial or financial relationships that could be construed as a potential conflict of interest.
